# What are the barriers to care integration for those at the advanced stages of dementia living in care homes in the UK? Health care professional perspective

**DOI:** 10.1177/1471301216636302

**Published:** 2016-03-01

**Authors:** Nuriye Kupeli, Gerard Leavey, Jane Harrington, Kathryn Lord, Michael King, Irwin Nazareth, Kirsten Moore, Elizabeth L Sampson, Louise Jones

**Affiliations:** Division of Psychiatry, Marie Curie Palliative Care Research Department, University College London, London, UK; Bamford Centre for Mental Health & Wellbeing, University of Ulster, Londonderry, UK; Division of Psychiatry, Marie Curie Palliative Care Research Department, University College London, London, UK; Division of Psychiatry, University College London, London, UK; Division of Psychiatry, University College London, London, UK; Department of Primary Care & Population Health, University College London, London, UK; Division of Psychiatry, Marie Curie Palliative Care Research Department, University College London, London, UK; Division of Psychiatry, Marie Curie Palliative Care Research Department, University College London, London, UK; Barnet Enfield and Haringey Mental Health Trust Liaison Psychiatry Team, North Middlesex University Hospital, London, UK; Division of Psychiatry, Marie Curie Palliative Care Research Department, University College London, London, UK

**Keywords:** integrated care, dementia, palliative care, qualitative research, care homes

## Abstract

People with advanced dementia are frequently bed-bound, doubly incontinent and able to speak only a few words. Many reside in care homes and may often have complex needs requiring efficient and timely response by knowledgeable and compassionate staff. The aim of this study is to improve our understanding of health care professionals’ attitudes and knowledge of the barriers to integrated care for people with advanced dementia. In-depth, interactive interviews conducted with 14 health care professionals including commissioners, care home managers, nurses and health care assistants in the UK. Barriers to care for people with advanced dementia are influenced by governmental and societal factors which contribute to challenging environments in care homes, poor morale amongst care staff and a fragmentation of health and social care at the end of life. Quality of care for people with dementia as they approach death may be improved by developing collaborative networks to foster improved relationships between health and social care services.

## Background

Dementia is a progressive, neurodegenerative disease. Currently, 850,000 people in the United Kingdom are living with dementia, and this figure will rise to over two million by 2051 (Alzheimer’s Society, 2014). A large proportion of the estimated 400,000 residents in UK care homes have dementia or another form of cognitive impairment (Care Quality Commission, 2010). Policy documents addressing dementia care are available in the UK and other countries including for example, USA and Australia (Ouwens, Wollersheim, Hermens, Hulscher, & Grol, 2005), but there is little attention to care at the end of life (Nakanishi et al., 2015).

People at the advanced stages of dementia may become doubly incontinent, unable to communicate their needs and often have multiple co-morbidities such as diabetes and hypertension (Gage et al., 2012). They are at increased risk of hospitalisation, following chest and urinary tract infections and frequently experience pain, anxiety and swallowing problems (Vandervoort et al., 2013). Despite these complex needs, people with advanced dementia often receive fragmented and suboptimal care at the end of life (Gage et al., 2012; Goddard, Stewart, Thompson, & Hall, 2013), and anticipation of future needs or care planning does not occur routinely. The recent White paper on the provision of optimal end of life care for people with dementia suggests that care for those approaching death should be provided by a multidisciplinary team (van der Steen et al., 2014).

Integrated care involves excellent communication between services, information sharing across disciplines and proactive anticipatory care, in particular management of symptoms and has been suggested to improve care outcomes (Wolfs et al., 2011). Integrated care models demonstrate improvement to both quality of care for those with chronic conditions (Ouwens et al., 2005) and quality of life of those at the early stages of dementia (Wolfs, Kessels, Dirksen, Severens, & Verhey, 2008). Importantly, people with advanced dementia should also have access to palliative care services (Lloyd-Williams, Abba, & Crowther, 2014), although research suggests that current palliative care teams may not be prepared for communication and behavioural problems presented by people with advanced dementia (Kupeli et al., 2016).

In the UK, the majority (53%) of those with dementia died in a long-term care institution in 2010 (Sleeman, Ho, Verne, Gao, & Higginson, 2014). In the UK, the National Health Service (NHS) allocates health and social care funding through local Clinical Commissioning Groups (CCGs). Clinical commissioners within CCGs are responsible for commissioning services that are lacking in their locality. Care homes are regulated by the Care Quality Commission (CQC), which is an independent organisation responsible for regulating and monitoring health and social care service providers to ensure the provision of care is safe, effective and of a high quality. The CQC are also responsible for ensuring that safeguarding procedures are adhered to by care homes. Safeguarding guidelines have been developed to ensure that vulnerable individuals are protected from harm and neglect.

However, care homes struggle to meet the needs of those who are approaching the end of life (Davies et al., 2014; Goddard et al., 2013). It has been suggested that palliative services are fragmented (Davies et al., 2014) and care homes are isolated within the wider network of services (Seymour, Kumar, & Froggatt, 2011). These issues are complicated by frequent reports that care home staff may have little skill in recognising when death is approaching and poor knowledge of medications commonly used to manage likely symptoms (Watson, Hockley, & Dewar, 2006). None of these studies, however, examine the issue of integrated palliative care within the care home setting from the perspective of those working both in care homes and provide support from external services.

### Aims

We aimed to identify the barriers to providing integrated care as understood by care professionals working with people with advanced dementia residing in care homes (all with some nursing beds). The work formed part of a larger national mixed methods programme to develop a complex intervention to improve end of life care for people with advanced dementia, as well as support those close to them.

## Methods

### Recruitment

We used purposive sampling to recruit Health Care Professionals (HCPs) across a range of organisations providing care for people with dementia. To gain a thorough and detailed understanding of HCPs experiences of providing care, we used a realist approach and in-depth interactive interviews (Pawson & Tilley, 1997). We interviewed a range of HCPs working for various organisations across North and South London including care homes and NHS services such as memory clinics, mental health and commissioning services. Interactive interviews enabled the interviewer and respondent to engage in a creative discussion without the restrictive parameters imposed by tightly structured interview schedules (Pawson & Tilley, 1997). Potential respondents were identified by research staff recruiting residents from care homes to a parallel cohort study (Sampson et al., 2016) and through the research team’s knowledge of other relevant HCPs. All respondents were sent a study information sheet and a reply slip to return to the researcher within two weeks. Those who returned the reply slip indicating their interest were contacted by research staff and appointments were made for interviews to take place at a location of the respondent’s choice, which for the majority of HCPs was at their place of employment. Consent forms were completed on the day of the interview after any questions had been answered. Ethical approval was granted by the University College London Ethics Committee (Reference: 3578/001) on 24^th^ January 2012.

### Data collection

Our preliminary topic guide was informed by emergent findings from a rapid literature review, workshops with health and social care professionals, carers and people with early dementia, and by information from an ongoing cohort study within the research programme (Sampson et al., 2016). We sought to explore service personnel’s recognition of dementia as a life-limiting illness, the management of acute medical problems, appropriateness of hospitalisation, the role of advance care plans and do not attempt resuscitation orders and HCPs views of where the health and social care system is failing to meet the needs of people with advanced dementia and their families. The researchers used emerging information to explore significant and unanticipated issues in later interviews.

### Data analysis

All interviews were audiotaped, transcribed and entered onto a qualitative software programme (Atlas-ti) for coding, management and retrieval of data. To ensure accuracy, two researchers checked the transcripts with the audio recordings. Transcripts were initially read several times to achieve familiarisation with the data. We analysed and coded the transcripts using thematic analysis as described by Braun and Clarke (2006). Units of text were broken down into categories to develop appropriate codes which were then grouped together to form themes. We used an iterative process to ensure that each unit of text was assigned to the correct code(s) and theme(s) as new categories emerged. For rigour and transparency, we used memos and reflective diaries. A third researcher reviewed three randomly selected interviews, and any differences in the coding framework were resolved through discussion. Minor grammatical errors in quotes have been corrected to facilitate comprehension.

## Findings

### Description of respondents

We interviewed 14 HCPs between September 2012 and October 2013. Respondents included three health-care assistants, one nurse, one clinical nurse manager, one clinical manager (manages a memory clinic service in an NHS-based organisation), two care home managers, one admiral nurse lead (specialist dementia nurse), one mental health nurse, one specialist mental health nurse and one occupational therapist. Two commissioners responsible for older adult services within a CCG were interviewed. While the range of experience and length of time in their current role (11 months to 14 years) was variable, all of the respondents were experienced in the health and social care sector. Interviews lasted approximately an hour.

### Barriers to integrated care

**Figure 1.**
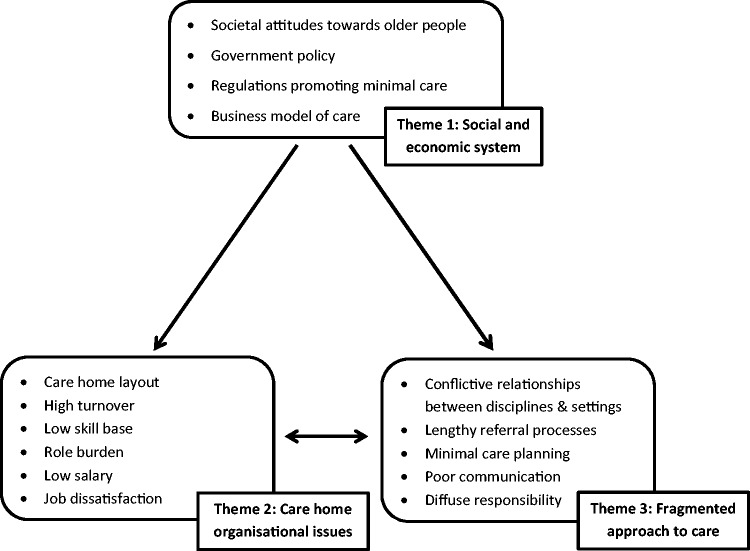
Barriers to integrated care for people with advanced dementia residing in care homes.

The data revealed three main themes consisting of several sub-themes; (1) social and economic system, (2) care home organisational issues and (3) a fragmented approach to care. A top-down hierarchical structure of how societal attitudes and the governmental system can influence the organisation within care homes and a fragmented approach to care is illustrated in Figure 1. The care home setting and fragmented approach to care also interact reducing the capacity of the care system to meet the end of life care needs of those with advanced dementia.

#### Theme 1: Social and economic system

The first theme is societal attitudes of older people and how the state develops services and implements policies related to the provision of care for people with dementia. Our data revealed that collectively as a society, we place less value on older people than on younger people, especially those nearing the end of life.

That would be like shoving me in a home when I get older and they've got me listening to dance music all the time. It's homogenous kind of 'one size fits all'… There was something like the King's report last year that said in society we're all so focused about the under threes and children, and actually the most vulnerable members in our society are older people, over the age of whatever it was. And I thought, 'Yeah, they are, because they just don't have a voice' (*Specialist Mental Health Professional*).

The commissioners were focused on issues related to the early stages of dementia rather than care in the later higher dependency stages of dementia.

So we have a number of dementia projects, business cases … for example to enhance the memory assessment service, and improve our early intervention and diagnosis … I think what we’re envisioning is some services will be key services … like the day service for people with dementia … a reading group (*Commissioner for Older Adults Services*).

There was consensus that UK Government strategy contributes to fragmented care. Limited funding and health and social care system reorganisation have been implemented to reduce costs, hence reducing the financial resources available for timely and coordinated care.

“I think now it's a big focus because of the viewing pathways, and looking at cost savings, and efficiencies,… it's financial priorities that we are expected to deliver on a big financial strategy” (*Commissioner for Older Adults Services*).

The data highlight a general concern by professionals that care homes are overly influenced by a business model of care, driven by profit rather than optimal care. Care homes can work around equivocal regulations such as the Health and Social Care Act 2008 (Regulated Activities) Regulations 2010 by assigning nurses to provide assistance to health-care assistants when staffing levels are at minimal levels.

By law it should be five to one [*ratio of care staff to residents*]. So the extra five, I chipped in most times to assist. And I work on the floor … [*Why do you think you’re understaffed at the moment?*] From my experience - its right across the whole country, you never get enough staff in. It’s only, occasionally you might have three carers to support you, but most of the time, the shortness of the adequate personnel is a crisis … Well, I do believe that one of the reasons is cost effectiveness. Not cost effectiveness - it’s got to do with finance. Not finance in the sense that, it’s [*care homes*] a profit making organisation, that doesn’t make it easy. And the less staff you get to carry out the task, the more profit you make (*Mental Health Nurse*).

#### Theme 2: Care home organisational issues

The physical layout of the care home may hinder the provision of good quality care. Many care homes are arranged so that people with different levels of need are housed on different floors of the building. Staff are required to work across these settings and the focus of their tasks may vary. Having staff consistently assigned to one floor was seen as promoting continuous care both to people with dementia and their families and enabled professional carers to adopt a more personalised approach.

I don’t know how confident the care home staff would have been to do it [*make referrals*] and also they changed on a daily basis, that was the biggest,… not the staff in the home but the floor that they worked on, yeah. So like you’d go do a ward round with (Doctor) and you’d sit there and you’d say ‘so how’s blah, blah been this week?’ and they’ll say ‘well I worked downstairs for the last five days then I had two days off’ and it would be like well what, you know we did say ‘please can you try and keep the same staff on the same floors’ and then you’d have RMN’s (*Registered Mental Nurse*) that worked on the middle floor which was more sort of general nursing and you would have an RGN (*Registered General Nurse*) on the bottom floor which was more psychiatric. There didn’t seem to be any consistency to be honest (*Clinical Manager*).

A strongly held view was that care homes were ill-equipped to provide adequate end of life care for residents with dementia. Due to the profit-driven nature of care homes, respondents reported that staffing levels were poor, and staff turnover was high due to limited professional development opportunities, demanding workloads, low pay and low job satisfaction resulting in high levels of role burden. Low availability of both trained and untrained staff resulted in demanding and stressful workloads forcing many staff to accept multiple responsibilities.

The workload also is extremely high for a nurse, for example the average amount of medications that a care home resident might be on may be 10, and that’s three times a day, they have to be given it at a certain time, the GP’s coming, the social worker’s doing a review, there’s a new admission, there’s a transfer, there’s a death, multiple, multiple things are happening at the same time that the nurse has to literally split herself in a hundred (*Care Home Manager*).

The less staff you get to carry out the task, the more profit you make … you get disgruntled. Why should I stretch myself etc … not that you don’t to want stretch yourself, but the impact of working endlessly has got a detrimental effect on your morale, output (*Mental Health Nurse*).

Care home staff were anxious about increasing governance and scrutiny that did not appear to take account of general working conditions, poor resources, challenging workload and the emotional demand of coping in these settings. Under the Department of Health’s Care Act 2014, safeguarding requires local authorities to make enquiries if abuse or neglect is suspected. Some staff felt that enquiries were sometimes made into issues that were not related to neglect and that the process of being investigated impacted on morale and created a sense of distrust.

Everything’s a safeguarding now, the bar is raised very, very high and we don’t see it as a negative, it is stressful when it happens in your home because you own your home and its, you think it’s a reflection on your home, and I think, I think the reason why it’s so stressful is that the social services raise safeguarding and then investigate … it’s not safeguarding if they didn’t change me within 5 or 10 minutes; it’s not a safeguarding. These are issues that we learn from. But already the flags are there, CQC (*Care Quality Commission*) is notified, and everything, everybody’s aware, and it just has a negative impact on the care in itself … It does have an impact. It does have an impact on morale (*Care Home Manager*).

Respondents reported that the commercial priorities of care homes negatively influenced staff career development leaving a considerable gap in training opportunities. These included training sessions on dementia, documentation completion, compassionate care and pain recognition. However, high staff turnover was a major obstacle to training. Conversely, lack of training may contribute to turnover.

[*if I could ask you to name a couple of top priorities that you think will improve care for this very vulnerable group of patients, particularly those approaching the end of their life, what do you think you'd say?*] Staff - really well trained staff. Really well trained staff. Of all the stuff that you've said, I think that well, well trained staff (*Specialist Mental Health Professional*).

There's such a high turnover of staff at that level [*care homes*], as you probably know. We have to do it [*training*] regularly, but we have to do some kind of toolkit which is really train the trainer with the toolkit, champions – it is a real challenge (*Commissioner for Older Adults Services*).

Respondents described how a low-skilled workforce hampered the potential of care home staff to deliver high-quality care resulting in poor symptom recognition and management. Care home staff argued that they were not provided with the training or support from external service providers to recognise and respond to symptoms presented by people with advanced dementia as they approached the end of life.

When someone says, ‘Oh, no, she’s not in pain,’ oh please - the nurses - ‘What do you mean she’s not in pain?’ They need to be more educated with dementia, because they can’t cope. There’s certain people that can cope with dementia, and a lot of people can’t cope with dementia. A lot of people do not like working with them, because they can’t cope with them. They don’t know how to respond to them (*Health Care Assistant*).

Some senior care home staff expressed frustration in the lack of motivation of team members and reported a poor uptake of recommendations by other staff. These opinions highlight the low skill base of care home staff, which appears to limit professionalism in role performance leading to problems within teams.

Some people will put some things in place but won’t think about everything else and its sort of left to me to do all the referrals and everything else. I’ll say have you done this, have you done that, have you done this, have you done that? (*Care Home Manager*)

It was suggested that care home staff appear demotivated in providing residents with anything more than basic care tasks, and consequently are perhaps unable to provide a more personalised and compassionate approach to residents dying with dementia.

“This is what I’m saying about carers in general, they won’t motivate their brain. They will do, they will wash, they will clean, they will feed, but not anything in between” (*Heath Care Assistant*).

#### Theme 3: Fragmented approach to care

Various thematic issues reflected fragmented care resulting from problematic relationships between care home staff and external HCPs. Care home staff contended that their knowledge and experience of caring for residents were under-recognised or devalued by other HCPs. Such issues deepen the apparent lack of trust between care home staff and service providers external to the care home.

She’s developed, rapidly developing grade four pressure sores everywhere, we’ve done safe guarding and everything else, she’s not on any analgesic but if you ask her she says she’s not in pain. So did a pain assessment day before, did one again yesterday and our GP was on holiday until today so trying to speak to another GP its sort of, ‘I want something for pain relief but she won’t take anything orally because she’s refusing all medication’, ‘well if she says not in pain from your pain assessment then I can’t prescribe something’, ‘yes but looking at her, she is in pain but she’s one of these people that sort of keeps quiet’, so it’s more about, I suppose people trusting you (*Care Home Manager*).

The processes implemented by care providers external to the care home, for example, lengthy referral processes and lack of advance care planning within primary and secondary care also resulted in uncoordinated and fragmented care.

[*is it easy to get other HCP in when needed?*] It’s not very easy … the social worker is okay, because you have the contact number and you can contact them but if you need the OT [*Occupational Therapist*] or physio or dietician or any other specialist who come from (Borough of London) reach you just fax over the referral but sometimes they can be very long because they have a long waiting list … And sometimes you need to re fax it to remind them because our report is getting through but they still do not get in contact with us. Sometimes two weeks pass and still nothing … (*Nurse*).

Information sharing was described as at its weakest when people with dementia were transferred between services, such as to hospital. In the quote below, a clinical manager laments the poor communication and the ill-defined responsibility when a patient is transferred to a care home from a psychiatric ward.

There’s absolutely zero information about the history and even the GP doesn’t have it either, you know, which I think is terrible. Yeah … Just crap communication yeah. And it’s up to the ward to ensure that information is, or although maybe it’s up to the placement officer, whoever decides on placing them to get that information but they’ve probably not got it either (*Clinical Manager*).

Care home staff described how hospital staff did not understand the nature and challenges that are faced by care homes. In the following quote, a care home manager points to ethical and procedural differences between residential care and hospital care, suggesting that residential care is negatively vilified. Again, such feelings are likely to result in further demoralisation in this sector and reduce integration of care.

When they go to the hospital from here, they always have a UTI, they’re always dehydrated, they have faecal impaction … And it’s ALWAYS, look that’s what’s happened, dehydrated. But none of this is true. What they [*hospital staff*] don’t understand is that we don’t force care here on anyone, for any reason whatsoever. We don’t restrain, we don’t restrain physically, chemically, we don’t put people in recliners that they can’t get up out of, that’s a restraint, we don’t open people’s mouths and give medication if they’re spitting it out, we don’t put fluids in people’s mouths if they don’t want to drink, the same thing with food, ok, and we have a balance that we, this is one of the stresses of the job in that when we admit somebody, and decide that we’re going to care for that person, we have a duty of care. We also on the other hand have to balance that with the person’s right to refuse … and I think, not just the hospital but I believe, social workers need to come in to care homes and I think they need to see for themselves what happens in care homes (*Care Home Manager*).

NHS-based services such as hospitals were described as having access to specialist resources and working in multidisciplinary teams in comparison to care home staff who work independently of other service providers.

“they [*care home staff*] work independently whereas in the hospital, if there’s a problem, call the doctor, call the physio” (*Care Home Manager*).

HCPs reported that they worked in silos, which hindered the development of an integrated care approach.

I still think there is, you know, they [*HCPs*] do work really well, but they do tend to work in silos, everybody still does work in silos. And it does need to be a bit more integrated. So integrated in terms of providers, and integrated across the pathway (*Commissioner for Older Adults Services*).

There was an element of diffused responsibility amongst providers of care for those with advanced dementia. Each expert is expected to provide care when their specialist advice is sought but a single health-care provider rarely takes overall responsibility for the health and well-being of those living in care homes.

We’ve got a single point of contact in this area so we’d refer via the single point of contact. For dieticians, and speech and language therapy we’ve got a form we fax off to the department. For palliative care we have to go through the GP and for podiatry we have to go through the GP (*Care Home Manager*).

## Discussion

To our knowledge, this is the first study to explore the barriers to providing integrated care to those with advanced dementia in care homes incorporating both HCPs working within and external to care homes. Given the growing population of people with dementia (Prince, et al., 2015) and that the majority of people with advanced dementia die in long-term care institutions (Sleeman et al., 2014), understanding and addressing the barriers to providing integrated care are critical. Our data suggest that three main factors hinder the development of an integrated approach to palliative care for those with advanced dementia: societal attitudes and governmental policy, the care home organisation and a fragmented approach to care. Within a youth-focused society, services for older people are given less emphasis and dementia care policy initiatives focus on assessment and the earlier phases of dementia.

Care home staff experience high role burden associated with demanding working conditions, very low pay and limited professional development opportunities. Our findings illustrate poor communication and conflictive relationships between HCPs and service settings, poor symptom management, lengthy referral processes, inter-agency ignorance, diffused responsibility amongst care providers and minimal care planning. These factors result in a fragmented rather than integrated approach to care.

Some of these findings are not unique to our study. Previous research highlights that tensions arise when other HCPs do not recognise that care home staff are familiar with their residents (Gage et al., 2012) and found disagreements regarding service responsibility (Jha, 2008). Limited professional development opportunities and low pay for formal carers in this sector are prevalent in countries other than the UK (Forbes & Neufeld, 2008). However, comparative work suggested that whilst health and social care services in the UK presented poor integration, care providers in the Netherlands functioned within collaborative networks (Kumpers, Mur, Hardy, van Raak, & Maarse, 2006). In addition, nursing homes in the UK have adopted a social care approach, whilst the Netherlands draws from a medical care model, thus enabling continuous access to doctors and other medical expert care for people with advanced dementia (Kumpers, Mur, Maarse, & van Raak, 2005) albeit at the cost of social needs.

The crucial need for integrated care, especially for those with advanced dementia has been highlighted by researchers in the US (Shega et al., 2003). Provision of proactive and integrated palliative care for people living with dementia in the community resulted in the rapid detection and management of symptoms and an increase in hospice referrals (Shega et al., 2003). Similarly, multidisciplinary teams caring for those with dementia score higher on quality indicators such as assessment and care planning, integration and provision of specialist care when compared with single health or social disciplines. However, the level of integration was not consistent across all multidisciplinary teams (Abendstern, Reilly, Hughes, Venables, & Challis, 2006) suggesting that coordinated care depends on individual teams at local levels. Similarly, UK studies indicate that collaboration at a local level is patchy and vulnerable to breakdown due to high staff turnover (Dening, Greenish, Jones, Mandal, & Sampson, 2012; Gage et al., 2012). While specialist palliative care from hospices could provide support to care homes, research shows these services may only be used in response to crises rather than as part of advance planning (Froggatt, Poole, & Hoult, 2002).

Business-driven care homes foster a culture of minimalistic provision of care aimed at meeting basic needs but neglecting, for example social interaction and emotional and spiritual support. A recent review found that these areas are just as important as ensuring that the physical needs of those at the end of life are fulfilled (Perrar, Schmidt, Eisenmann, Cremer, & Voltz, 2015).

Lack of professional development opportunities for care home staff also contributes to the cycle of poor provision of fragmented care. A recent review highlighted that providing structured training programmes to formal carers working in care homes can improve behavioural problems presented by people with dementia and reduce the use of antipsychotic medication (Fossey et al., 2014). Integration of care should be available continuously throughout the disease and not just at the diagnostic stage (Bullock, Iliffe, & Passmore, 2007; McNulty, Jackson, & Pelosi, 2008).

### Strengths and limitations

Our study has a number of strengths including the recruitment of commissioners and HCPs from a variety of services, the collection of rich data from in-depth interactive interviews and the use of triangulation and consensus meetings to verify the credibility of the data. Our realist approach to developing the topic guide informed by our findings from the other components of the programme ensured that the interviews addressed issues that were emerging directly from our work with services.

Although the study did not aim to be representative of all HCPs, data were collected from HCPs working in care homes and those providing care to care homes in the South East of England. However, these attitudes may not be applicable to other HCPs such as general practitioners and allied health professionals and those working in other areas of the UK or in other countries. Finally, an integrated approach to providing end of life care for people with advanced dementia requires collaboration between care providers and family carers. Although it is a limitation that we have not included family carers perspectives, we did seek their views on this topic and hope to publish these findings separately.

### Implications

Our findings have implications for research, policy and practice. They support two of the top 10 research priorities for care at the end of life identified by the Palliative and end of life care Priority Setting Partnership (PeolcPSP) (2015) which worked in consultation with people with dementia, their family carers and HCPs providing care for those with palliative care needs. This report stresses the need for training to be provided to HCPs delivering palliative care and research to develop a continuous model of care.

We suggest that integration of care would be developed by modifying structural factors and the network of services that currently function as separate entities when providing care for people with dementia. The Better Care Fund (National Health Service (NHS) England, 2013) proposed by the UK Government could help reduce the fragmentation in the provision of good end of life care for those with dementia, for example, by developing networks and shared referral pathways to foster improved relationships between health and social care providers.

Our finding that care homes operate on minimal staffing levels is not surprising as the Health and Social Care Act 2008 (Regulated Activities) Regulations 2010 is open to interpretation stating that care services must employ “sufficient numbers of suitably qualified, skilled and experienced persons.” This should be replaced with definitive recommendations on the optimal ratio of HCPs to residents in care homes to ensure good care. In addition, good practice could be incentivised by rewarding care homes who adhere to such recommendations as part of standard practice.

## Conclusion

Care of people with dementia who are approaching death may be enabled by an integrated approach that includes improving the communication and relationships between all care providers and creating multidisciplinary teams who draw on each other’s knowledge to provide optimal care. For this, a top-down approach from policy makers is required so that the funding and resources are available to develop leaders in this field who will motivate all care providers to work together at local level. Successful development of an integrated model could result in an environment and culture where older people are more valued and cared for by a proactive, collaborative team of experts who provide health and social care.
